# Scale-Specific Multifractal Medical Image Analysis

**DOI:** 10.1155/2013/262931

**Published:** 2013-08-19

**Authors:** Boris Braverman, Mauro Tambasco

**Affiliations:** ^1^Department of Physics, MIT-Harvard Center for Ultracold Atoms and Research Laboratory of Electronics, Massachusetts Institute of Technology, Cambridge, MA 02139, USA; ^2^Department of Physics, San Diego State University, 5500 Campanile Drive, San Diego, CA 92182-1233, USA

## Abstract

Fractal geometry has been applied widely in the analysis of medical images to characterize the irregular complex tissue structures that do not lend themselves to straightforward analysis with traditional Euclidean geometry. In this study, we treat the nonfractal behaviour of medical images over large-scale ranges by considering their box-counting fractal dimension as a scale-dependent parameter rather than a single number. We describe this approach in the context of the more generalized Rényi entropy, in which we can also compute the information and correlation dimensions of images. In addition, we describe and validate a computational improvement to box-counting fractal analysis. This improvement is based on integral images, which allows the speedup of any box-counting or similar fractal analysis algorithm, including estimation of scale-dependent dimensions. Finally, we applied our technique to images of invasive breast cancer tissue from 157 patients to show a relationship between the fractal analysis of these images over certain scale ranges and pathologic tumour grade (a standard prognosticator for breast cancer). Our approach is general and can be applied to any medical imaging application in which the complexity of pathological image structures may have clinical value.

## 1. Introduction 

Many biological phenomena exhibit chaotic or fractal-like behavior, and these features have been studied extensively [1–5]. One area of widespread use has been the application of fractal geometry in the analysis of medical images as it lends itself naturally to the pragmatic characterization of irregular non-Euclidean structures found in medical imaging [6–9]. However, care must be taken when applying fractal analysis to natural objects [10]. The quintessential requirement for an object to be a fractal is for the object to exhibit a form of self-similarity to arbitrarily small scales. As such, actual fractals do not exist in nature, since there is a fundamental natural limitation to the scaling behaviour of natural objects when their substructures approach the atomic scale. Even real renderings of mathematical fractals cannot be truly fractal because of the finite resolution of the rendering. In general, the scaling behaviour of natural objects depends on the scale at which the objects are considered. Despite this limitation, most fractal analysis techniques have focused on characterizing the fractal behaviour of natural objects by finding an interval of scales in which these objects have an approximately constant scaling behaviour [3].

Some authors have noted the danger in making this interpretation because of the possibility of illusory “fractal” behaviour where none is actually present [11], as well as the difficulty in defining the interval of scales over which the object has a consistent scaling behaviour [12]. To limit these difficulties, our approach in recent studies was to manually pick the interval of scales based on physical considerations (i.e., range of the sizes of the histological structures of interest) and the linearity of the dependence of entropy on scale [13–16].

Another approach used by others to resolve this difficulty has been to model the scale dependence of the fractal dimension in a rendering of a mathematical fractal by fitting a functional model to the dimension as a function of scale and interpreting the fit parameters as meaningful fractal dimensions for the renderings [17, 18]. The conceptual arguments behind these models are derived for renderings of mathematical fractals, and they may not necessarily hold for images of natural objects. However, the authors of these studies are aware that natural objects do not fit the mold of real mathematical fractals.

In this study, we adopt the view expressed by others that the fractal dimension of a natural object changes with scale. Assuming that physically meaningful information in an image is contained in the scaling behaviour of the image in a certain interval of spatial dimension, then a thorough and objective approach to extract this information would be to assess the clinical relevance of different scale ranges and determine an absolute scale at which the fractal dimension is relevant for a given medical imaging application. That is, the spatial scale interval chosen for the analysis of medical images would be selected on the basis of patient classification performance and patient outcome for diagnostic and prognostic applications, respectively, rather than the resemblance of the image structures to a fractal at these image scales.

In order to address the shortcomings of existing techniques, we propose an approach to analyzing the scaling behaviour as a function of scale itself, providing an estimate of the fractal dimension as a scale-dependent parameter rather than a single fixed value. We present this fractal analysis approach in the context of the more generalized Rényi entropy [19], in which we can also compute the information and correlation dimensions of images [20].

In addition, we describe a novel fractal analysis algorithm based on integral images [21] which speeds up the computation of these generalized dimensions by orders of magnitude, and we validate our algorithm and approach by applying it to increasingly complex data, producing meaningful results throughout. We also illustrate our method with the analysis of a set of histology images acquired from tissue samples of breast tumours with modified Bloom-Richardson grades of 1, 2, and 3, in which we determine the spatial scale intervals that exhibit statistically significant differences in fractal dimensions between the different tumour grades. Looking forward, this approach provides a rapid tool to determine an absolute scale at which the generalized fractal dimensions are relevant and may allow for objective interimage comparison of medical images acquired at different resolutions.

## 2. Fractal Dimension

Fractal dimension (FD) is a generalization of the intuitive notion of topological dimension and allows for noninteger dimensions; for example, a point, a line, and a plane have both topological and fractal dimensions of 0, 1, and 2, respectively. However, fractal objects such as the Koch snowflake and Sierpinski carpet both have a topological dimension of 1 but have fractal dimensions of 1.26 and 1.89, respectively. The fractal dimension of an object is fundamentally a reflection of its scaling behaviour. Hence, to provide an estimate of the fractal dimension of a real image, we first need to define a scale-dependent function *f*(*A*, *ϵ*), with pixel intensities *A*(*x*, *y*) defined for (*x*, *y*) ∈ *S*, where *S* is the area contained in the image and *ϵ* is the analysis scale. For mathematical fractals, the fractal dimension is usually defined as

(1) $$\begin{array}{c} D\equiv -\underset{\epsilon \to 0}{\lim}\frac{f\left(A,\epsilon \right)}{\log\left(\epsilon \right)}. \end{array}$$

However, for a real image it is meaningless to analyze an infinitely small scale by letting *ϵ* → 0, and so at any given scale we can define the fractal dimension as

(2) $$\begin{array}{c} D\left(\epsilon \right)\equiv -\frac{\partial f\left(A,\epsilon \right)}{\partial \log\left(\epsilon \right)}. \end{array}$$

The function *f*_*b*_(*A*, *ϵ*) corresponding to the box-counting dimension is commonly used in medical imaging because of its conceptual and algorithmic simplicity. In this method, the parameter *ϵ* is the side length of the square boxes into which the image is partitioned, and *f*_*b*_(*A*, *ϵ*) is the logarithm of the number of squares of this size in the image that contains at least one pixel that is a part of a structure of interest.

### 2.1. Generalized Dimensions

The box-counting function *f*_*b*_(*A*, *ϵ*) is a special case of a more generalized class of functions, the Rényi entropies [19], which also generalize Shannon entropy. The Rényi entropy *H*_*α*_ of order *α* of a probability distribution {*μ*_*i*_} is given by

(3) $$\begin{array}{c} H_{\alpha }\equiv \frac{1}{1-\alpha }\log\left(\sum_{i}\mu_{i}^{\alpha }\right). \end{array}$$

The values {*μ*_*i*_} are the set of natural measures of the probability distribution. For an image data set *I*(*x*, *y*) and a scale *ϵ*, the values {*μ*_*i*_} are found by dividing the image into *ϵ* × *ϵ* squares, and for each square by finding the proportion *μ*_*i*_ of the total image intensity that is contained in the *i*th square:

(4) $$\begin{array}{c} \mu_{i}\equiv \frac{\int_{S_{i}}I\left(x,y\right)}{\int_{S}I\left(x,y\right)}, \end{array}$$

where *S*_*i*_ is the domain of the image contained in the *i*th square. Definition (4) implies ∑_*i*_ *μ*_*i*_ = 1 because the set *S* is entirely covered by the subsquares *S*_*i*_.

The fractal dimensions *D*_*α*_ = −lim⁡_*ϵ*→0_ *H*_*α*_/log⁡(*ϵ*) obtained by using different orders *α* generate the *multifractal spectrum* [6] of an image. There are three values of *α* that have clear physical significance [22]. When *α* = 0, we treat the term *μ*_*i*_^*α*^ in (3) as the limit of *μ*_*i*_^*α*^ as *α* → 0. Hence, for *μ*_*i*_ ≠ 0, *μ*_*i*_^0^ = lim⁡_*α*→0_ *μ*_*i*_^*α*^ = 1, while for *μ*_*i*_ = 0, *μ*_*i*_^0^ = lim⁡_*α*→0_ *μ*_*i*_^*α*^ = 0. The entropy *H*_0_ is therefore the logarithm of the number of nonzero values of *μ*_*i*_ in the image, which is the same as logarithm of the number of nonempty squares of size *ϵ* in the image, equaling the box-counting function *f*_*b*_ from before. The Rényi entropy of order 0 will thus yield the *box-counting dimension D*_0_ of the image.

If *α* = 1, we again treat (3) as a limit when *α* → 1. In this case, by using L'Hôpital's rule for the limit as *α* → 1, the Rényi entropy reduces to the Shannon entropy as follows:

(5) $$\begin{array}{c} H_{1}\left(\epsilon \right)=-\sum_{i}\mu_{i}\log\left(\mu_{i}\right). \end{array}$$

The dimension *D*_1_ obtained from the Shannon entropy is known as the *information dimension* of the image. This measure of the fractal dimension gives the rate at which information is gained about the structure of the image as the resolution of the image increases.

The third value of *α* with a clear physical meaning is *α* = 2, which gives rise to *D*_2_, the *correlation dimension* of the image, addressing the number of neighbours a point of the structure has as a function of scale. That is, *D*_2_ gives the power law that relates the number of other image pixels that are within a range *ϵ* of a given pixel to the value of *ϵ*.

In essence, larger *α* values assign a greater weight to the brighter parts of the image being analyzed. This is particularly useful for the analysis of medical images in which both the spatial structure and relative intensity of edge structures may carry useful information about the image.

## 3. Methods and Materials

### 3.1. Scale Dependence of Fractal Dimension

As mentioned in the introduction, natural objects do not exhibit true fractal behaviour, as their scaling behaviour depends on the scale at which the objects are considered. Hence, we propose an objective approach to determine the scale or scale interval in which the different orders of fractal dimensions may have physical relevance (e.g., diagnostic or prognostic value in medical imaging).

To estimate the fractal dimension *D*_*α*_(*ϵ*) as a function of scale, we use definition (2) and measure the entropies *H*_*α*_(*ϵ*) given by (3). To differentiate the entropy with respect to the analysis scale, we use the locally weighted regression and smoothing scatterplots (LOWESS) method [23], which is widely used in situations where a good theoretical model for the observed data does not exist. A low-order polynomial is fitted to a weighted subset of the data around each data point, and then all parameters (such as the derivatives) of the fitted curve can be extracted from this polynomial. In our case, we are interested in the first derivative of the entropy with respect to scale, which immediately gives a scale-dependent estimate of the fractal dimension of the image. The advantage of this method over direct numerical differentiation is its vastly enhanced robustness to noise, while its advantage over a fit to the entropy or numerical derivative data, as used by others [17, 18], is in its greater flexibility and scale resolution, as well as a lack of assumptions that may not be valid for images of natural objects.

### 3.2. LOWESS Method

To estimate parameters of a scatterplot of a data set *Y* = {*y*(*x*)} given as a function of *X* = {*x*} using the LOWESS method [23], for each *x*_0_ ∈ *X*, we fit a polynomial *p* to the data in such a way as to minimize the weighted sum of the squared residuals *R* given by *R* = ∑_*x*∈*X*_(*y*(*x*) − *p*(*x*))^2^ · *w*_*x*_0__(*x*), where *w*_*x*_0__(*x*) is a weighting function. The fitted curve *f*(*x*) is then approximated around *x*_0_ by the polynomial *p*. In particular, for *x* ≈ *x*_0_, we estimate the *n*th derivatives of *f* and *p* as equal for *n* ≤ deg⁡(*p*).

The function *w* is central to the LOWESS method, since it makes the regression *locally* weighted. The simplest weighting function *w* that can be used makes the fit around a point *x*_0_ local a Gaussian with a standard deviation *σ*:

(6) $$\begin{array}{c} w_{x_{0}}\left(x\right)=\exp\left[-{\left(\frac{x-x_{0}}{\sigma }\right)}^{2}\right]. \end{array}$$

Larger values of *σ* will smooth out the fit more than smaller values, producing a fit that is more resistant to noise at the cost of resolution in *x*. In the case of scale-dependent fractal analysis, the input data *x* and *y* to the LOWESS model are *x* = log⁡(*ϵ*) and *y*(*x*) = *H*_*α*_(*ϵ*), from which we can obtain *D*_*α*_(*ϵ*) = −*f*′(*ϵ*).

### 3.3. Rapid Computation of the Natural Measures

The most common operation encountered in the box-counting approach to fractal image analysis is the determination of the natural measure *μ*_*i*_ given in (4) of a certain rectangular (usually square) subset *S*_*i*_ of the image. If the subset *S*_*i*_ is bounded by *x*_1_ < *x* < *x*_2_ and *y*_1_ < *y* < *y*_2_, while the entire image is bounded by 0 < *x* < *x*_*M*_ and 0 < *y* < *y*_*M*_, finding the natural measure is equivalent to calculating

(7) $$\begin{array}{c} p_{i}=\int_{S_{i}}I\left(x,y\right)dx\;dy=\sum_{\begin{array}{c} x_{1}<a\leq x_{2}\\ y_{1}<b\leq y_{2} \end{array}}A_{a,b}, \end{array}$$

(8) $$\begin{array}{c} P=\int_{S}I\left(x,y\right)dx\;dy=\sum_{\begin{array}{c} 0<a\leq x_{M}\\ 0<b\leq y_{M} \end{array}}A_{a,b}, \end{array}$$

(9) $$\begin{array}{c} \mu_{i}=\frac{p_{i}}{P}. \end{array}$$

**Figure 1:**
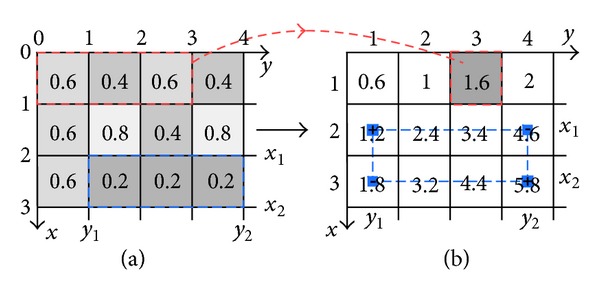
An illustration of an intensity image (a) being summed to produce an integral image (b). The sum of the elements of (a) in the dotted red box gives the corresponding element of (b). After (b) is computed, the sum of the elements of (a) in the dashed blue box, with 2 < *x* < 3, 1 < *y* < 4, can be found by using (11). In this particular example, the sum equals 0.6, while *B*_3,4_ − *B*_3,1_ − *B*_2,4_ + *B*_2,1_ = 5.8 − 4.6 − 1.8 + 1.2 also equals 0.6.

For example, in Figure 1, the sum of the image intensity *p*_*i*_ in image *A* over the dotted (red) rectangle bounded by 0 < *x* < 1 and 0 < *y* < 3 is equal to *p*_*i*_ = *A*_1,1_ + *A*_1,2_ + *A*_1,3_ = 0.6 + 0.4 + 0.6 = 1.6.

Direct summation of the image intensity over a rectangle has computational cost proportional to the area of the rectangle. A method to speed up the summation is to first compute the following *integral image* (also known as the *summed area table*) [21]:

(10) $$\begin{array}{c} B_{x,y}=\sum_{\begin{array}{c} 0<a\leq x\\ 0<b\leq y \end{array}}A_{a,b}. \end{array}$$

This computation reduces the subsequent summation in (7) to a simple arithmetic operation (e.g., see Figure 1):

(11) $$\begin{array}{c} p_{i}=\sum_{\begin{array}{c} x_{1}<a\leq x_{2}\\ y_{1}<b\leq y_{2} \end{array}}A_{a,b}=B_{x_{2},y_{2}}-B_{x_{1},y_{2}}-B_{x_{2},y_{1}}+B_{x_{1},y_{1}}, \end{array}$$

where we define *B*_*x*0_ = 0 and *B*_0*y*_ = 0 for all *x* and *y*.

The usefulness of this algorithm lies in its ability to speed up natural measure computation. The computational complexity of a naïve algorithm, in which for each scale *ϵ* we determine the natural measures, (4), by directly summing the image intensity is of order *O*(*A* · *s*), where *A* is the area of the image and *s* is the number of different values of *ϵ* considered. However, by using the integral image algorithm approach, the computational complexity falls to *O*(*A*) since the cost of computing the natural measures at a scale *ϵ* is approximately equal to *A*/*ϵ* ^2^ and ∑_*ϵ*=1_^*∞*^1/*ϵ* ^2^ = *π* ^2^/6 ≈ 1.6 is small. The integral image approach is particularly advantageous for large values of *ϵ* where a large number of summations are replaced with only a few subtractions. For a typical fractal scale analysis, the integral image approach is 10 to 100 times faster than the naïve algorithm.

### 3.4. Algorithm

Our method was implemented in MATLAB Version 7 (The MathWorks, Inc., Natick, MA, USA). The steps of the method are as follows.

(i) Select the analysis scales *ϵ* and entropy order *α* values. The choices made are based on purely physical considerations, for example, the range of sizes of the structures of interest in the image being analyzed. To be general, one can begin by analyzing a wide range of *ϵ* and *α* values and select the physically meaningful subset of scales and orders.
(ii) Normalize the original image *A* by dividing every element by the sum of the pixel intensities of the entire image, giving the natural measure contained in each image pixel.
(iii) Calculate the integral matrix *B* for the normalized image *A*. For each scale *ϵ*, rescale the image using the integral matrix. That is, calculate the matrix
  (12) $$\begin{array}{c} R_{x,y}=\sum_{\begin{array}{c} i=(x-1)\epsilon +1\\ j=(x-1)\epsilon +1 \end{array}}^{\begin{array}{c} i=x\epsilon \\ j=y\epsilon \end{array}}A_{ij}. \end{array}$$
(iv) For each value of *α*, raise the natural measure matrix *R* to the power *α* except for *α* = 1 obtaining *M*_*ij*_ = *R*_*ij*_^*α*^; for *α* = 1, we calculate the matrix *M*_*ij*_ = −*R*_*ij*_ · log⁡(*R*_*ij*_).
(v) Use (3) and (5) to give *H*_*α*_ = (1/(1 − *α*))log⁡(∑*M*_*ij*_) for all *α* ≠ 1, while for *α* = 1, *H*_1_ = ∑*M*_*ij*_.

Our method was tested on the following data sets: exact renderings of deterministic strictly self-similar fractals, randomized renderings of deterministic fractals, randomized renderings of statistically self-similar fractals, and a set of breast histological tissue samples.

### 3.5. Breast Cancer Tissue Specimens

 In a previous study [16], we had retrospectively selected 408 patients with primary invasive ductal carcinoma (IDC) of the breast from Calgary regional hospitals after appropriate ethics approval from the institutional review board. The breast tissue from these patients was used to construct tissue microarray (TMA) cores (each with a 600 *μ*m diameter) stained with pan-cytokeratin to highlight the morphology of epithelial architecture. The number of cores per patient ranged from one to three. Images of the cores were acquired with an effective magnification of 6.3x using an AxioCam HR digital camera (Carl Zeiss, Inc.) mounted on an optical microscope (Zeiss Axioscope). The images were saved at the camera's native resolution of 1300 × 1030 pixels in tagged image file format (tiff). In this previous study [16], we found the box-counting fractal dimension of the breast cancer TMA core images to be an independent and statistically significant prognosticator. However, the study did not include an explicit examination of the role of the scale range on the fractal dimensions computed from the images. Instead, fractal dimension was computed from plots of the slope of *f*_*b*_(*A*, *ϵ*) versus log⁡(*ϵ*) over a scale range of (*ϵ* = 10–50 *μ*m), which was chosen based on a visual assessment of the range of the linear region of a small random sample of plots taken from the whole image set.

**Figure 2:**
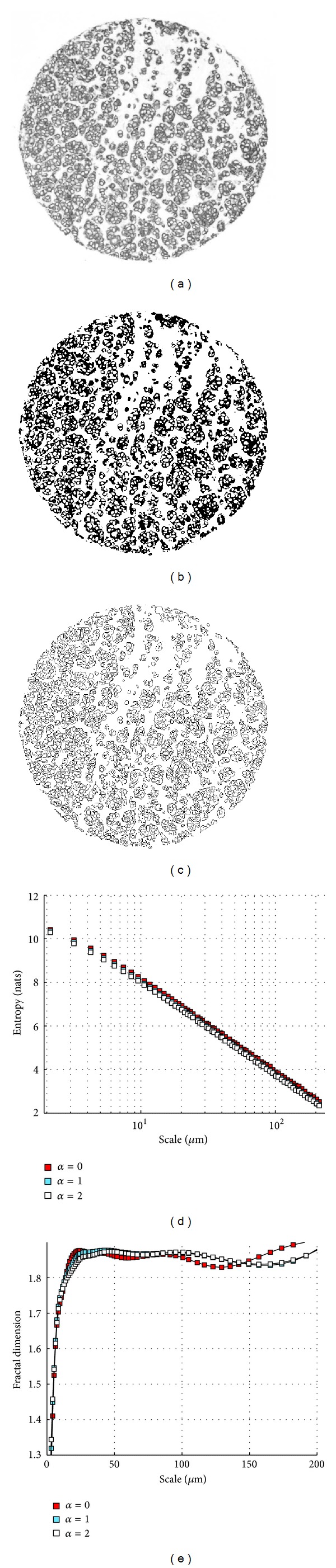
An illustration of the overall analysis process. (a) Grayscale image of a breast tissue sample (600 *μ*m in diameter). (b) Black and white thresholded version of (a). (c) Outlines of (b). (d) Image entropies determined from (c). (e) Scale-dependent fractal dimensions for this tissue sample.

In this study, we selected all the cases from our previous study set of 408 patients [16] that satisfied the conditions of having exactly three evaluable TMA core images and contained pathologic grade information. This selection resulted in a set consisting of a total of 157 patients in the following tumour grade categories: 56 grade 1, 84 grade 2, and 17 grade 3 tumours. We applied our method to these cases, and the analysis was used to demonstrate the capabilities of the algorithm and to check that our previous choice for the scale range was a judicious one. The grayscale images of the tissues were converted into black-and-white outline images by thresholding. An example of the overall analysis process for a breast cancer tissue core is shown in Figure 2. To determine the optimal thresholding level for the edge detection, we varied the threshold level for each image to maximize the fractal dimension in the 10–50 *μ*m scale interval.

The motivation for using three cores per patient (chosen from different tumour regions) was to ensure that we had a representative sample of a heterogeneous tumour. For the final analysis, we selected the one core from each patient that had the greatest average fractal dimension in the 10–50 *μ*m scale range. The rationale for choosing the core with the greatest fractal dimension is that it is likely representative of the portion of a possibly heterogeneous tumour that has deviated most from normal epithelial breast morphology, and therefore it is the most probable indicator of abnormal and/or aggressive tumour growth with metastatic potential. For the analysis, we produced a single curve of fractal dimension as a function of scale *ϵ* for each patient. We averaged the fractal dimensions within each tumour grade category.

### 3.6. Statistics

We performed the statistical analysis using the MATLAB Statistics Toolbox 7.4 (The MathWorks, Inc., Natick, MA, USA). We quantified the differences between the three tumour grade categories using the nonparametric Kruskal-Wallis analysis and a multiple comparison test (MATLAB functions kruskalwallis and multcompare, resp.).

## 4. Results

### 4.1. Assessment of Scale-Dependent Algorithm on Renderings of Strictly Self-Similar Fractals

**Figure 3:**
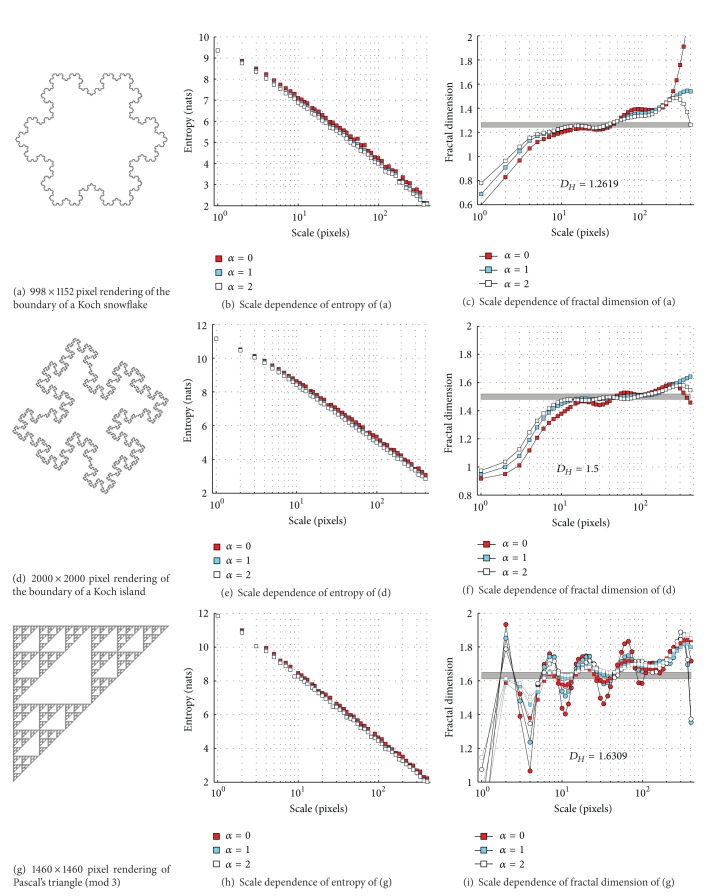

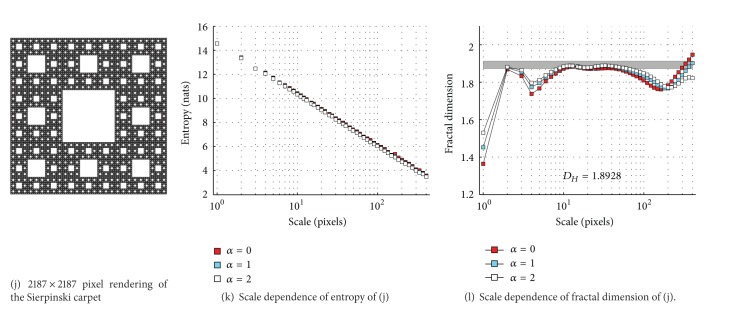
Results of applying our algorithms to four renderings of mathematical fractals. The entropies in (b), (e), (h), and (k) are in nats, which are the natural units for information and entropy, with base *e* rather than 2: 1 nat ≈ 1.44 bits, and are plotted for *α* = 0,1, 2. All the fractal dimension plots (c), (f), (i), and (l) use *σ* = 0.5 in (6), except for (i) where *σ* = 0.3 for the circles. Horizontal bar indicates Hausdorff dimension *D*_*H*_ of each mathematical fractal.

Figure 3 shows the results of our approach applied to renderings of strictly self-similar mathematical fractals. For the Koch snowflake, Pascal triangle, and Sierpinski carpet, the estimated fractal dimensions are consistently within 0.1 of the Hausdorff dimension of the mathematical fractal being rendered throughout the scale range of 10 to 250 pixels. Note that both the mean and maximum deviations of the measured fractal dimension from the Hausdorff dimension decrease with increasing fractal dimension, increasing value of *σ* in (6), and have a minimum in the range of 20 to 50 pixels, where neither the small nor large-scale granularities of the image affect the estimate of the fractal dimension. In this smaller interval of scales, the measured fractal dimensions have a root-mean-squared deviation of 0.074, 0.040, 0.086, and 0.029 for *σ* = 0.3 and 0.045, 0.018, 0.052, and 0.034 for *σ* = 1.0 from the Hausdorff dimension, respectively, for the four fractals presented.

For the Koch island boundary (Figure 3(d)), a jump of fractal dimension from 1 to 1.5 (the Hausdorff dimension) can be seen at *ϵ* ≈ 4 pixels. This behaviour is consistent with the real structure of the rendering, which at small scales consists of straight line segments of 4–8 pixels long. Hence, below a scale of 4 pixels, the rendering is really linear and hence has a fractal dimension of 1 at this scale.

In Figure 3(i), we compare the estimate of fractal dimensions produced by using the values of 0.3 and 0.5 for *σ* in (6). The larger value of *σ* produces a smoother dependence of fractal dimension on scale, which is close to the Hausdorff dimension of Pascal's triangle. On the other hand, the smaller value gives an estimate which is more locally accurate, showing the nonuniform scaling behaviour of the rendering, which can be seen in the oscillations in the fractal dimension as a function of scale. In fact, this inhomogeneous scaling behaviour is seen in all four of the sample renderings. These oscillations occur because the real renderings of the fractals have discrete characteristic scales. For example, the Pascal's triangle mod 3 rendering (Figure 3(g)) consists of black and white triangles of several discrete scales: 3, 9, 27, 81, 243, and 729 pixels. Around each of these scale values, the fractal dimension experiences a large drop. This drop occurs because when the analysis scale grows through each of these scales, the white triangles in the rendering become “invisible” to the box-counting algorithm, causing a smaller than expected drop in the entropy *H*_*α*_ and consequently causing a dip in the box-counting dimension. However, between these characteristic scales, the image becomes nearly 2-dimensional, just like the plane in which the image is contained, because the analysis cannot “see” any change in the image features.

### 4.2. Assessment of Scale-Dependent Algorithm on Renderings of Statistical Fractals

We further tested our algorithm on two kinds of statistical fractals, which are generated by random processes, but nonetheless possess a statistical form of self-similarity.

#### 4.2.1. Randomized Sierpinski Triangle

**Figure 4:**
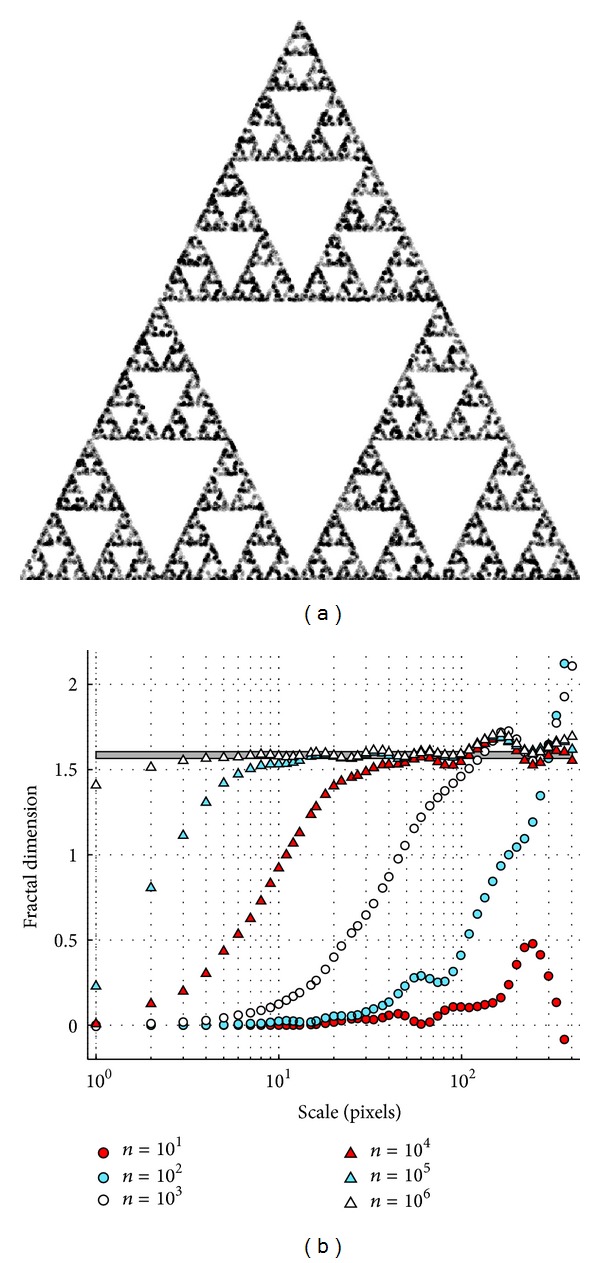
(a) 2000 pixel × 2000 pixel rendering of a statistical Sierpinski triangle (10^4^ points). Each point in the statistical fractal is rendered as a small grey disk for clarity. (b) Scale dependence of (box counting) fractal dimension of statistical Sierpinski triangles rendered with the indicated number of points (legend). The value *σ* = 0.3 is used in (6). Horizontal bar shows Hausdorff dimension *D*_*H*_ = log⁡(3/2) ≈ 1.585.

An approximation to a Sierpinski triangle can be generated by an iterative random process known as the “chaos game” [24]. In each step of the game, one new point is added to the rendering of the triangle. We employed this method with a variable number of iterations (from *n* = 10 to *n* = 10^6^) to generate several Sierpinski triangle approximations, with an example for 10000 points shown in Figure 4(a). When the structure is rendered with only a few points, the structure of the randomized triangle is essentially point-like or 0-dimensional at small scales. For example, with only 1000 points in a 2000 × 2000 pixel image, the expected mean distance between points is $2000/\sqrt{8n}=23$ pixels; indeed, the fractal dimension measured for *n* = 1000 begins to grow around that scale, but already has the Hausdorff dimension log⁡(3/2) scaling behaviour at larger scales above 100 pixels.

In this case, we can see that our algorithm correctly identifies the scaling behaviour of the fractal renderings: the large-scale behaviour of the fractal quickly reaches the correct scaling power law, while the smaller-scale features are really 0-dimensional. However, as more and more points are added to the approximation, the fine-scale structure of the fractal is filled in, and the fractal dimension approaches its ideal value at all scales.

#### 4.2.2. Brownian Surfaces

**Figure 5:**
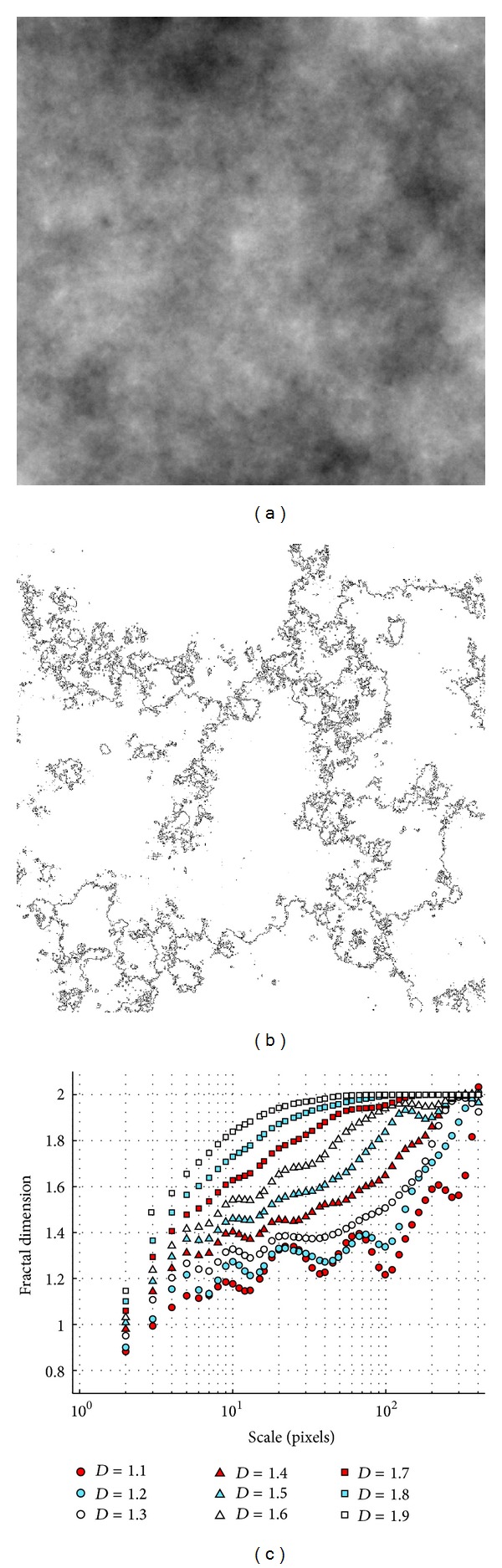
(a) 1025 × 1025 pixel rendering of a Brownian surface of dimension 2.5. (b) Outlines of (a), which have a theoretical fractal dimension of 2.5 − 1 = 1.5. (c) Scale dependence of fractal dimension of (a) along with 8 other Brownian surfaces of different fractal dimensions. The theoretical dimensions of the outline images are indicated in the legend.

The fractal dimension of nine 1025 × 1025 pixel renderings of Brownian surfaces with dimensions 2.1 through 2.9 were evaluated by finding cross-sections through the images (which should have a dimension that is one dimension less than the surfaces themselves). The results of this analysis are shown in Figure 5. Several features can immediately be seen from Figure 5(c). All of the outlines are inherently 1-dimensional at small scales because of their linear structure. The Brownian surface outlines (i.e., Figure 5(b)) pack the image more densely as the scale of the image increases, causing the fractal dimensions to approach 2 for all the cross-sections. These features are *not* due to a flaw in the algorithm, but rather reflect the true behaviour of the curves obtained by slicing through the Brownian surfaces. The behaviour of other measures of fractal dimension, such as other components of the multifractal spectrum, is similar to the box-counting dimension shown in the figure. The fractal dimensions match the expected values most closely in the 10–40 pixel range, with a root-mean-square error of less than 0.06 in this interval. This example illustrates the great sensitivity of fractal dimension measures to the scale at which they are computed and the consequent need for a scale-dependent measure of the fractal dimension to quantitatively estimate this sensitivity and choose an appropriate scale or scale range for analysis.

### 4.3. Application of Scale-Dependent Method to Breast Tissue Samples

**Figure 6:**
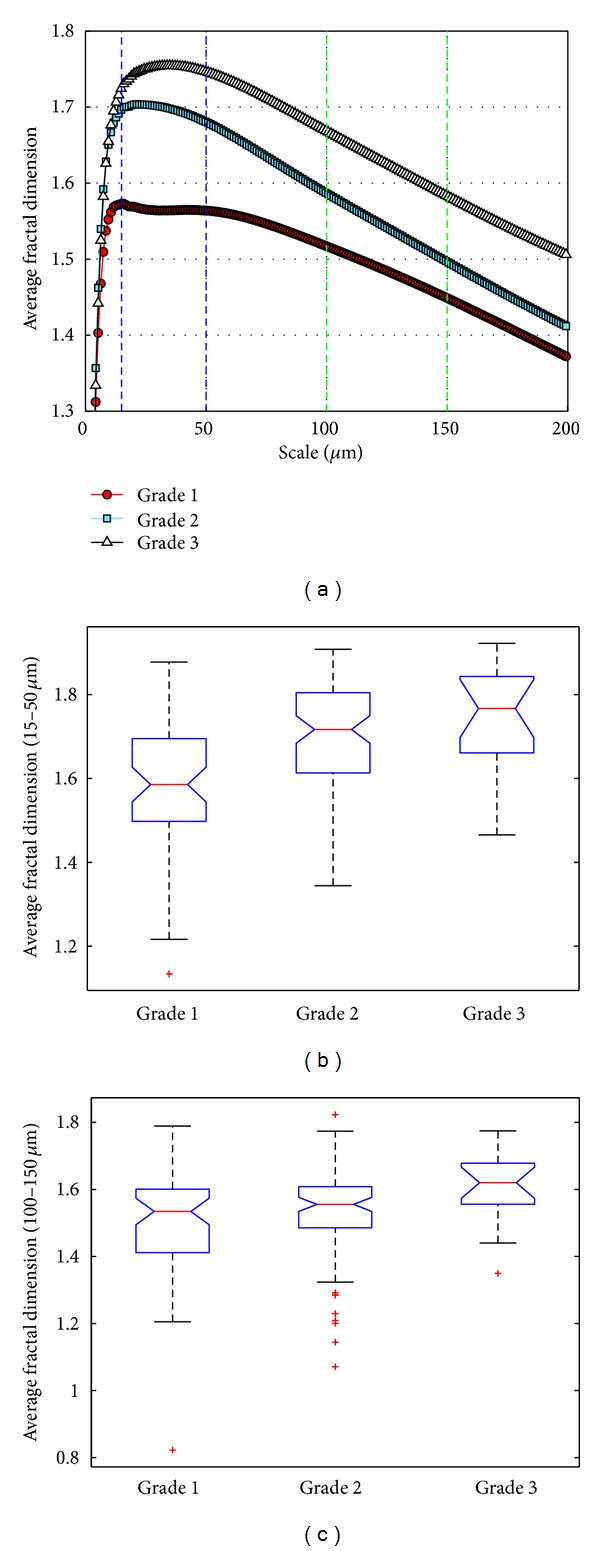
(a) Fractal dimensions of breast histology images (averaged over all images in each grade) as a function of the image scale. The two scale intervals (15–50 *μ*m and 100–150 *μ*m) used as examples are indicated by the vertical dashed lines. (b), (c) Boxplots of fractal dimensions in the scale interval 15–50 *μ*m and 100–150 *μ*m, respectively. See main text for statistical analysis.

It is readily apparent that the curves of the averaged fractal dimensions within each tumour grade category are similar at small scales below 5 *μ*m but rapidly differentiate at larger scales Figure 6(a). In addition, there seems to be a larger difference between grades 1 and 2 at smaller scales and a larger difference between grades 2 and 3 at larger scales.

The Kruskal-Wallis analysis showed that statistically significant differences exist between at least 2 of the 3 tumour grade groups for fractal dimensions averaged over scale ranges of 15–50 *μ*m (*P* < 0.0001) and 100–150 *μ*m (*P* < 0.008). The multicomparison test showed that a statistically significant difference (*P* < 0.0005) exists between grades 1 and 2 and 1 and 3 in the 15–50 *μ*m scale range and a statistically significant difference (*P* < 0.05) between grades 1 and 3 and 2 and 3 in the 100–150 *μ*m scale range (Figure 6(a)). Figures 6(a), 6(b), and 6(c) also show the fractal dimension distributions for the different grades and scale ranges in the form of boxplots. For these plots, the middle 50% of the data lie within the boxes, the lines within the boxes are the median values, the lines above and below the boxes show the upper and lower 25 percent of the data, respectively, and the crosses outside the boxes show outliers. The results illustrate the way scale range can affect the results and how different ranges can be useful for distinguishing different groups (e.g., grades 2 and 3 were not significantly different in the smaller 15–50 *μ*m scale range, but differences become more significant in the 100–150 *μ*m larger-scale range).

In a previous study using a scale range of 10 to 50 *μ*m, it was found that FD < 1.56, 1.56 < FD < 1.75, and FD > 1.75, correlated to high, intermediate, and low survival from breast cancer [16]. The results of this study are consistent with the previous finding, as higher tumour grade is also correlated to poorer survival, and grades 1, 2, and 3 correspond to similar FD ranges (Figure 6). It is important to note that any comparisons with other results reported in the literature will only be meaningful if a similar scale range is used for finding the fractal dimension and if the tissues' specimens are stained using the same stain and tissue preparation technique.

## 5. Conclusion

In this study, we have described two novel ideas for the application of fractal analysis to medical images: fractal dimension as a scale-dependent scaling parameter of a statistical distribution and an application of the integral image method for rapid evaluation of fractal dimensions. The notion of considering fractal dimension as a model-free scale-dependent parameter is a fundamental shift in perspective on investigations of fractal image analysis. By forgoing a specific model for how an image should behave, we allow ourselves to extract as much information as possible from the image. Hence, the true power of our method lies in its ability to determine the appropriate scale range or ranges that need to be analyzed using fractal methods for any particular application. In testing our algorithms on both real fractal structures and medical images, we showed the algorithm's reliability in measuring fractal dimensions and in picking up subtle scale-dependent features in the fractal dimensions. More specifically, our analysis of invasive breast cancer tissue cores from 157 patients has shown that the ability to differentiate images of different grades of cancer depends on the scale at which images are analyzed. As tumour grade is a prognosticator for breast cancer survival, it is evident that the analysis scale has an impact on the prognostic value of a fractal analysis approach, a point which has not been systematically studied or appreciated previously. Future studies are needed to further validate and extend the breast cancer results using an independent set of tissue images.
